# Numerical Responses of *Xylocoris flavipes* (Reuter) (Hemiptera: Anthocoridae) on a Diet of *Liposcelis decolor* (Pearman) (Psocodea: Liposcelididae)

**DOI:** 10.3390/insects16030296

**Published:** 2025-03-12

**Authors:** Augustine Bosomtwe, George Opit, Carla Goad, Kristopher Giles, Brad Kard

**Affiliations:** 1 Department of Entomology and Plant Pathology, Oklahoma State University, 127 Noble Research Center, Stillwater, OK 74078, USA; george.opit@okstate.edu (G.O.); kris.giles@okstate.edu (K.G.); b.kard@okstate.edu (B.K.); 2 CSIR-Plant Genetic Resources Research Institute, Bunso P.O. Box 7, Ghana; 3 Department of Statistics, Oklahoma State University, 301 Mathematics, Statistics and Computer Sciences, Stillwater, OK 74078, USA; carla.goad@okstate.edu

**Keywords:** warehouse pirate bug, psocid, biological control, stored-product pest management, biocontrol agent

## Abstract

Insect predators can suppress prey populations through increased progeny production. The present study investigated the numerical responses of adult♀ *Xylocoris flavipes* (Reuter) (Hemiptera: Anthocoridae) on a diet of *Liposcelis decolor* (Pearman) (Psocodea: Liposcelididae). Adult♀ *X. flavipes* were placed in arenas containing nymphs, adult females, or males of *L. decolor* at varying prey densities under laboratory conditions at 28 ± 1 °C, 63 ± 5 RH, and a 0:24 (L:D) photoperiod. The number of eggs laid by the predator was assessed at 24 h intervals for five days, and this was used to determine oviposition rate, oviposition efficiency, and efficiency of conversion of ingested food resources (ECI). This study showed that prey stage did not affect oviposition rate; however, there was a positive correlation between prey density and *X. flavipes* oviposition rate for all the prey stages. The predator’s oviposition efficiency and ECI (%) were inversely proportional to prey density for all prey stages. The current study shows that *X. flavipes* can produce more offspring on adults and nymphs of *L. decolor* when prey densities are high and can establish at low prey densities. Further evaluation of *X. flavipes* under field storage conditions is recommended to facilitate its incorporation into integrated management of psocids.

## Simple Summary

Stored-product psocids are difficult to manage with phosphine, an effective insecticide against many stored-product pests. A common predator associated with insect pests and mites in storage environments is the warehouse pirate bug, *Xylocoris flavipes*, which has potential for use as a biocontrol agent in stored-grain psocid pest management. The aim of this study was to provide data on the numerical responses of adult♀ *X. flavipes* on a diet of nymphs and adults of *Liposcelis decolor* by assessing the predator’s oviposition rate, oviposition efficiency, and efficiency of conversion of ingested food resources (ECI). This study showed that adult♀ *X. flavipes* has high oviposition rates when prey densities are high. The relationship between oviposition efficiency and prey density was inversely proportional, as was the relationship between ECI and prey density. The high predation rate indicates the potential of *X. flavipes* for effective psocid management. By increasing predator progeny production with prey density, *X. flavipes* showed that it can numerically respond to psocid population dynamics. *X. flavipes* also demonstrated the ability to establish readily at lower prey densities.

## 1. Introduction

Over the last four decades, psocids (Psocodea: Liposcelididae) have emerged as an economically important insect pest of stored-products [1,2]. Among several species of psocids known to infest stored-products, *Liposcelis decolor* is one of four species reported as economically significant pests worldwide [2]. Psocids are capable of causing significant weight losses by consumption of germ and endosperm of stored grain kernels [3,4]. Infestation of food commodities by psocids, including *L. decolor*, can lead to rejection during trade [2]. Economically important psocid species are difficult to manage with phosphine, the most commonly used and usually effective insecticide against coleopteran and lepidopteran pests due to natural tolerance and rapid development of resistance [2,3,5]. Laboratory studies have shown that certain psocid species, including *Liposcelis decolor* (Pearman) and *Liposcelis entomophila* (Enderlein), can tolerate relatively high concentrations of phosphine up to 249.76 and 697.29 ppm or 194.5 and 157.1 ppm over a 20 h or 72 h fumigation period, respectively [6]. The surge of psocids control failures in many countries has been attributed to the development of phosphine resistance and the greater tolerance of eggs to phosphine [2]. For instance, psocid populations have been found to recover much more rapidly from poorly applied phosphine fumigations than beetle pests do, and they benefit from the reduction in predators and competitors, including the red flour beetle [2].

The potential of using natural enemies, including the predatory warehouse pirate bug, *Xylocoris flavipes* (Hemiptera: Anthocoridae), as an additional method of managing stored-product insect pests has been reported in several studies [7,8,9]. *Xylocoris flavipes* is distributed worldwide in storage and processing facilities and has the natural ability to penetrate grain masses. It has been approved for use against stored-product insect pests in the United States [10,11,12]. Biological control agents target specific pest species, reducing reliance on broad-spectrum insecticides. This approach is less harmful to humans and the environment, is sustainable, and can serve as an additional method for insect pest control in a domain where there are limited available insecticides, and insect pests are becoming more resistant to existing synthetic insecticides [7].

One of the density-dependent behaviors by which predators respond to prey populations is numerical response, which describes the change in predator population density through mechanisms, including reproduction, survival, or both due to changing prey density [13]. Numerical response provides information on resource utilization, reproductive output, and colonization potential of predators [14,15,16]. Insect predators consume several prey in the course of development, but there is not a single universal relationship between the number of prey consumed in one generation and the number of predators in the next generation [17]. However, several studies have demonstrated an increase in predator numbers following a rise in prey density [16,18,19,20]. Female predators use numerical response as a mechanism to optimize offspring production depending on prey availability [16,21].

In numerical response models, ways in which prey availability can affect the birth, death, and dispersal rates of predator populations are primarily considered. It is known that many aspects of insect biology are embedded in a nutritional context, and parental nutrition affects oviposition, size of eggs, and quality in terms of protein content [22,23]. An insect predator that develops in favorable conditions of abundant and nutritious prey, optimal temperature, relative humidity, and photoperiod can reach its maximum physiological potential, resulting in a high reproductive capacity [23]. Generally, nutrition and its dependence on prey density produce a numerical response in a predator population [17].

*Xylocoris flavipes* is known as an efficient predator of eggs, larvae, and pupae of beetles and moths [7,8]. A recent study reported that both adult♀ and nymphs of *X. flavipes* prey on mobile life stages (nymphs and adults) of *L. decolor* and therefore have the potential to manage stored-product psocids [9]. However, there is little to no information on the reproductive capacity of *X. flavipes* when on a diet of psocids. Natural enemies, including parasitoid wasps and predatory mites, are able to produce progeny when feeding on psocid pests [16,24,25,26,27]. Therefore, *X. flavipes* may also be capable of augmenting its progeny production at varying prey densities to suppress psocid populations in stored commodities.

Considering this potential, the objective of the present study was to assess the numerical responses of the adult♀ *X. flavipes* on a diet of nymphs or adults of *L. decolor*. The predator’s efficiency of conversion of ingested food resources (ECI) was also evaluated. The ECI shows the relationship between the conversion of prey biomass and prey density [28].

## 2. Materials and Methods

### 2.1. Rearing of Liposcelis decolor

*Liposcelis decolor* used as prey in this study were reared on a psocid diet as described in [16,27]. *Liposcelis decolor* was used as a diet for rearing *X. flavipes*. Additionally, *L. decolor* nymphs and adults from the established cultures were selected and used for this study. Cultures of *L. decolor* were maintained for at least three generations in the laboratory before they were used for this study.

### 2.2. Rearing of Xylocoris flavipes

Laboratory stock cultures of *X. flavipes* were initially obtained from Biologische Beratung GmbH, Berlin, Germany. Colonies of *X. flavipes* were subsequently maintained on *L. decolor*. Cultures of *L. decolor*, which were approximately 4 weeks old and had been established in plastic boxes (75 ± 5% RH) inside a growth chamber maintained at 30 ± 1 °C and a 0:24 (L:D) photoperiod, were used to start *X. flavipes* cultures. Temperature and relative humidity during this study were monitored using onset^®^ HOBO^®^ Data Loggers (LI-COR Environmental, Lincoln, NE, USA). Approximately 50 pairs of *X. flavipes* were introduced into the psocid jars to feed on *L. decolor*. The jars containing both *X. flavipes* and *L. decolor* were placed in plastic boxes (42 × 29 × 24 cm high) painted black, which contained saturated NaNO_2_ solution (sodium nitrite, anhydrous, free-flowing, Redi-DriTm, ACS reagent, ≥99%, 746,398–2.5 KG, Sigma-Aldrich, Inc., St. Louis, MO, USA) beneath perforated false floors to maintain 63 ± 2% RH. The plastic boxes were then placed inside a growth chamber and maintained at 28 ± 1°C and a 16:8 (L:D) photoperiod for the *X. flavipes* to multiply and establish. Given that the warehouse pirate bug is cannibalistic, cultures were frequently monitored, and *L. decolor* were added biweekly to prevent decline in predator populations because of starvation or conspecific predation. Stock cultures of *X. flavipes* were maintained for at least three generations in the laboratory before they were used for this study.

### 2.3. Experimental Arenas

Experimental arenas consisted of a 5.0 cm diameter basal Petri dish covered by a 5.5 cm diameter lid (forming a total cylindrical surface area of 54.98 cm^2^; the total migration surface area for a predator in a cylinder of one basal Petri dish and a lid) (50 × 10 mm and 55 × 10 mm Style Polystyrene, Falcon^®^, Becton Dickinson and Company, Franklin Lakes, NJ, USA). The experimental arenas were prepared as described in [16,27], with 29.99 cm^2^ as a total migration area of prey.

### 2.4. Numerical Responses of Adult♀ X. flavipes

Five- to eight-day-old adult females (hereafter referred to as adult♀) were selected from pure cultures of *X. flavipes* and were starved for 24 h prior to being placed in arenas containing their prey, that is, nymphs, adults (males or females) of *L. decolor* at varying densities. Adult♀ *X. flavipes* of this age, which were assumed mated, were selected because of the preoviposition period (the time between adult emergence and oviposition of the first eggs), which was at least four days. Starvation decreased oviposition, standardized their level of hunger, and initiated a nomadic period [3,29,30]. Experimental arenas with adult♀ *X. flavipes* had different densities of the specific prey stage. Prey densities of 2, 6, 15, 30, 40, 50, 60, 70, and 80 were transferred, as described in [16,27]. Each prey stage × prey density combination was replicated six times in this study for the determination of numerical responses of adult♀ *X. flavipes*. Each of the six replications was run separately at different times. This means each replication had all 3 prey stages and 9 prey densities. Culture jars from which predators and prey were collected and used for each replicate were different (that is, insects in a particular jar or set of jars were used for only one replicate). For each replicate where adult♀ *X. flavipes* were evaluated, diets of nymphs or adults (males or females) of *L. decolor* at the 9 prey densities were used according to the method described in [16,27]. Thus, 1 predator stage × 3 prey stages × 9 prey densities were run as a replicate each separate time slot for a total of 6 replications. For each replication set consisting of 27 experimental arenas (3 × 9), all arenas were arranged randomly in a single plastic box (42 × 29 × 24 cm high) painted black, which contained saturated NaNO_2_ solution beneath perforated false floors to maintain 63 ± 2% RH, and a box was kept inside a growth chamber maintained at 28 ± 1 °C and a 0:24 (L:D) photoperiod. Arenas were assessed at 24 h intervals to count the number of eggs laid by adult♀ *X. flavipes* and the number of prey killed using the method described in [16,27]. For each prey stage, a total of 54 (1 prey stage × 9 prey densities × 6 replications) adult♀ *X. flavipes* were assessed in the 6 replications. Data on each batch of replicates were collected over 120 h (5 days), and five data sets per adult♀ *X. flavipes* were recorded at 24 h intervals. Thus, data for each replicate represent five 24 h observations. Therefore, for 6 replications of each prey stage, there were 270 observations (54 × 5) in total. The average of the daily number of eggs laid (*N_o_*) and the average of the daily number of prey consumed (*N_a_*) at the end of the fifth consecutive data collection were assumed to represent the daily predator oviposition rate and predation rate, respectively, of adult♀ *X. flavipes* in their confined arenas at a specified prey density (*N*). Data collected were used to model the numerical responses of adult♀ *X. flavipes*. Additionally, per capita oviposition rate (eggs/day), per capita oviposition efficiency (*N_o_*/*N*), and per capita ECI (%) were estimated for each adult♀ *X. flavipes*. The efficiency of conversion of ingested food resources (ECI) was calculated using the equation ECI = [(*N_o_*/*N_a_*) × 100] at different *N* [19]. The parameter ECI quantifies the relationship between the conversion of prey biomass and prey density [28]. The experimental setup was a Completely Randomized Design (CRD) replicated 6 different times.

### 2.5. Statistical Analysis

The numerical responses of adult♀ *X. flavipes* when on a diet of nymphs and adults of *L. decolor* were evaluated based on regression models to determine the relationship between oviposition and prey density. The polynomial y = a + bx + cx^2^ was selected to fit the data for adults of *L. decolor*, whereas the linear y = a + bx equation was fitted for the nymphs of *L. decolor* based on p values and coefficients of determination (R^2^). The regression models were fitted using R version 4.3.2 (R Core Team 2023).

The mean number of eggs laid (*N_o_*), the oviposition efficiency (*N_o_*/*N*), and the efficiency of conversion of ingested food (ECI) were compared across the 3 prey stages and 9 prey densities using generalized linear mixed model methods. The model included the main effects of prey stage and prey density and their interaction for each of the response variables (*N_o_*, *N_o_*/*N*, and ECI). The mean number of eggs laid was modeled using a Poisson distribution, and oviposition efficiency and ECI were modeled as an exponentially distributed random variable. All tests were conducted at the nominal 0.05 level of significance. Least squares means were compared for the appropriate significant effects using the Tukey method. All data were analyzed using SAS software Version 9.4 (SAS Institute Inc., Cary, NC, USA) and R version 4.3.2 (R Core Team 2023).

## 3. Results

### 3.1. Number of Eggs Laid by Adult♀ Xylocoris flavipes

**Figure 1:**
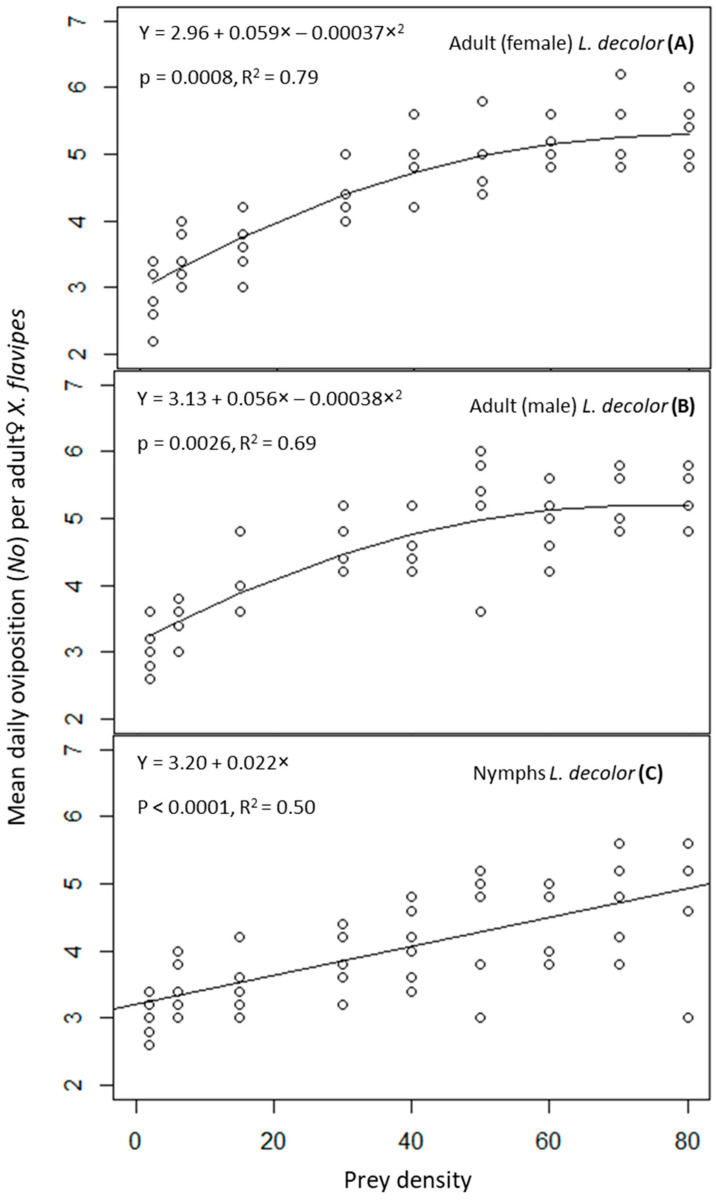
Oviposition rate of adult♀ *Xylocoris flavipes* on a diet of adult female (**A**), or adult male (**B**), and nymphs (**C**) of *Liposcelis decolor* at varying densities.

The interaction of prey stage and prey density on numbers of eggs laid (*N_o_*) by adult♀ *X. flavipes* was not significant (*p* > 0.05) (Table 1). However, prey density was significant (*p* ≤ 0.05), but prey stage was not significant (*p* > 0.05) (Table 1). Mean numbers of predator eggs laid significantly increased with increasing prey density, and this numerical response was best described by a polynomial function for adults of *L. decolor* and a linear function for the nymphs of *L. decolor* (Figure 1). Generally, higher numbers of predator eggs were laid at prey densities of 60, 70, and 80. This was approximately 1.5 times the numbers laid at prey densities of 2 and 6. For prey densities of 15, 30, 40, 50, 60, 70, and 80, adult♀ *X. flavipes* on a diet of adults of *L. decolor* numerically produced slightly more eggs compared with when feeding on nymphs, although the differences were not statistically significant (Table 2).

### 3.2. Oviposition Efficiency of Adult♀ Xylocoris flavipes on a Diet of Liposcelis decolor at Different Developmental Stages and Densities

In relation to oviposition efficiency, the interaction of prey stage and prey density was not significant (*p* > 0.05) (Table 1). However, prey density was significant (*p* ≤ 0.05), but prey stage was not significant (*p* > 0.05). There was a decrease in oviposition efficiency with increasing prey density. Oviposition efficiency of the lowest prey density (2) was the highest with values of 1.47 ± 0.60, 1.53 ± 0.63, and 1.53 ± 0.63 for adult females, adult males, or nymphs of *L. decolor*, respectively, compared with approximately 0.07 recorded at the highest prey density of 80 for all prey stages (Table 3).

### 3.3. Efficiency of Conversion of Ingested Food Resources (ECI) of Adult♀ Xylocoris flavipes on a Diet of Liposcelis decolor at Different Developmental Stages and Densities

Similar to oviposition efficiency, the interaction of prey stage and prey density was not significant (*p* > 0.05) in relation to ECI (Table 1). Prey density was significant (*p* ≤ 0.05), but prey stage was not significant (*p* > 0.05). There was an inverse relationship between ECI and prey density. The ECI values for the lowest prey density of 2 were the highest across all *L. decolor* stages. The mean percent ECI values were 149.07 ± 60.86, 153.33 ± 62.60, or 156.11 ± 63.73 for adult females, adult males, or nymphs of *L. decolor*, respectively, at a density of 2; these ECI values were approximately 12–13 times higher than those for prey densities above 50 (Table 4).

## 4. Discussion

Insect predator populations respond to changes in prey density either by a change in the number of prey killed by each predator or a change in the density of predators’ population through reproduction, survival, or both [13]. These responses are among the diverse mechanisms insect predators, including anthocorids and predatory mites, use to suppress pest populations [9,16,23,27,31,32]. The present study investigated the numerical responses of the predatory warehouse pirate bug, *Xylocoris flavipes*, on a diet of *Liposcelis decolor*, a psocid species with high tolerance to phosphine [2,6]. The oviposition rate of adult♀ *X. flavipes* correlates positively with prey density, and prey stage did not affect oviposition rate. Oviposition of insect predators positively correlates with prey density because females may allocate a major fraction of food ingested to egg production [33]. The increase in oviposition rate in response to the increase in prey density suggests that *X. flavipes* can respond to increasing psocid populations in storage environments by exerting greater pest suppression through its own population growth and hence has potential for effective management of *L. decolor*. Predators displaying strong density-dependent reproduction rates are considered promising biocontrol agents because of their capacity to suppress pest populations before reaching damaging levels [34]. Although total egg production increased with prey density, the rate was slower than the increase in prey density, indicating that *X. flavipes* may not convert all captured and killed (attacked) prey into egg production. This means the predator kills prey wastefully or defensively rather than to enhance reproductive success [27,35]. Density-dependent wasteful killing, where some prey items are partially consumed, is known to be both an adaptive foraging and aggression strategy used by predators to suppress pest populations [35,36]. In a similar study by Danso et al. [16], oviposition in the predatory mite, *Cheyletus eruditus* (Schrank) (Trombidiformes: Cheyletidae), was negatively correlated with prey density in the case of adults of *L. decolor*, but positively correlated for nymphs. For *Cheyletus malaccensis* Oudemans, the authors [16] found a positive correlation between oviposition and prey density in the case of all the different developmental stages of *L. decolor*. They [16] also found that in these two predatory mites, oviposition efficiency was negatively correlated with prey density in the case of all of the different developmental stages of *L. decolor*. Female predators use numerical response as a mechanism to optimize offspring production depending on prey availability [16,21]. *Xylocoris flavipes* is known to be an efficient predator of stored-product beetles and moths and requires low prey numbers for complete development [37]. A study by Brower and Press [38] showed that residual populations of several species of small beetles in empty grain bins were greatly reduced by 70 to 100% during weekly releases of 50 pairs of *X. flavipes*. In a related study, a combination of *X. flavipes* and parasitoid *Theocolax elegans* showed a synergistic effect in controlling *Rhyzopertha dominica* (Fabricius) (Coleoptera: Bostrichidae) and *Sitophilus oryzae* (L.) (Coleoptera: Curculionidae) [39]. Bosomtwe et al. [9] reported that both adults and nymphs of *X. flavipes* attack all mobile stages of *L. decolor,* indicating the predator’s potential for control of psocids. It has a high capacity to increase in population relative to its prey and destroys large numbers of prey when abundant [7,9,37,39]. For rapid suppression of psocid populations, augmentation by inundative release of *X. flavipes* can, for example, be deployed in warehouses containing bagged commodities, animal feed or pet food bagging areas, empty storage structures, and places where pallets are stored. However, to target early stages of psocid infestation when pest density is low, it would be efficient to implement inoculative releases of *X. flavipes* [16,40].

Oviposition efficiency plays a critical role in regulating predator–prey population dynamics and contributes to the success of biological control [41]. Prey consumption and predator egg production efficiency influence predator population growth rates and their capacity to respond numerically to high pest populations [19]. The current study showed that oviposition efficiency of *X. flavipes* exhibited an inverse relationship with prey density. A similar inverse density-dependent oviposition efficiency was observed in *C. eruditus* and *C. malaccensis* when attacking *L. decolor* [16]. This could be a physiological or adaptive mechanism that optimizes resource allocation at low prey density and can be advantageous in practical biocontrol scenarios, as it enables predator populations to persist in low pest density scenarios and then to respond quickly when pest populations resurge [19,33,42].

The ECI reveals the relationship between the conversion of prey biomass and prey density [28]. Factors, including prey quality, stage, sex, and prey type, influence ECI [43]. In this study, ECI decreased substantially with increasing prey density across the different prey stages. Higher predator ECI at low prey density and subsequent decrease at higher prey densities have been reported in several studies [16,19,28]. At low prey density, adult♀ *X. flavipes* are likely to focus their energy on egg production and consequently invest less in maintenance and metabolic activities [19]. However, at higher prey density, the predator may engage in defensive and wasteful killing of prey rather than utilizing most of the attacked prey for oviposition [35]. The decreasing efficiency of prey conversion into eggs with increasing prey density is known to be consistent with predators that exhibit type II functional response [44]. Although higher oviposition efficiency was observed at lower prey densities, the oviposition rate was low. Therefore, estimating an optimal prey density that optimizes ECI and increases oviposition rate should be considered in the context of IPM for stored-product psocids. There were no differences in ECI when *X. flavipes* consumed different *L. decolor* developmental stages. Similarly, *C. malaccensis* showed no considerable differences in ECI when on diets of different developmental stages of *L. decolor* [16]. In Danso et al. [16], ECI for *C. eruditus* was considerably higher at lower prey density of male adult psocids but showed a general trend of decreasing ECI with increasing prey density in the case of all the different developmental stages of *L. decolor*.

## 5. Conclusions

The current study showed that adult♀ *X. flavipes* can prey on *L. decolor* to survive and establish and can produce significant numbers of offspring to suppress and stabilize *L. decolor* populations. The findings suggest that *X. flavipes* may be most effective as a biological control agent when released prophylactically or early in pest infestation when psocid densities are relatively low due to the predator’s increased resource utilization at low prey density. Based on the results of this study, prey developmental stage did not significantly influence oviposition rate, oviposition efficiency, or efficiency of conversion of ingested food resources. Because predator–prey population dynamics are influenced by several abiotic and biotic factors, further studies on other foraging behaviors such as mutual interference, prey switching, and the role of spatial complexity in predator–prey population dynamics are required.

## Figures and Tables

**Table 1:** Summary of the tests for the main effects of prey stage (PS) and prey density (*N*) of *Liposcelis decolor* on numbers of eggs laid (*N_o_*), per capita oviposition efficiency (*N_o_*/*N*), and per capita efficiency of conversion of ingested food resources (ECI) of adult♀ *Xylocoris flavipes*.

Predator	Variable	Source	Df	*F*	*p*–Value
Adult♀ *X. flavipes*	*N_o_*	PS	2, 135	0.60	0.5492
		*N*	8, 135	2.29	0.0247
		PS*N	16, 135	0.05	1.0000
	*N_o_*/*N*	PS	2, 135	0.15	0.8630
		*N*	8, 135	20.72	<0.0001
		PS*N	16, 135	0.01	1.0000
	ECI	PS	2, 135	0.02	0.9782
		*N*	8, 135	14.42	<0.0001
		PS*N	16, 135	0.01	1.0000

**Table 2:** Mean number of eggs laid (*N_o_*) (mean ± SE) per adult♀ *Xylocoris flavipes* per day on a diet of *Liposcelis decolor* at different developmental stages and densities.

Predator	Prey Density (*N*)	Eggs Laid per Predator (*N_o_*)		
		Prey Stage		
		Female	Male	Nymph
Adult♀ *X. flavipes*	2	2.93 ± 0.67 B	3.07 ± 0.72 B	3.07 ± 0.71 B
	6	3.53 ± 0.77 AB	3.50 ± 0.76 AB	3.53 ± 0.77 AB
	15	3.63 ± 0.78 AB	4.00 ± 0.82 AB	3.50 ± 0.76 AB
	30	4.40 ± 0.86 A	4.70 ± 0.89 A	3.80 ± 0.80 AB
	40	4.90 ± 0.90 A	4.67 ± 0.88 A	4.10 ± 0.83 AB
	50	4.83 ± 0.90 A	4.93 ± 0.91 A	4.43 ± 0.86 A
	60	5.10 ± 0.92 A	4.97 ± 0.91 A	4.37 ± 0.85 A
	70	5.33 ± 0.94 A	5.20 ± 0.93 A	4.80 ± 0.89 A
	80	5.30 ± 0.94 A	5.30 ± 0.94 A	4.87 ± 0.90 A

Significant differences among numbers of eggs laid per predator at different prey densities in the case of each prey stage diet are denoted by different letters (*p* ≤ 0.05, SAS, Tukey’s Honestly Significant Difference Test). There were no significant differences among numbers of eggs laid for diets of different prey stages in the case of each prey density.

**Table 3:** Per capita oviposition efficiency (*N_o_*/*N*) (mean ± SE) of adult♀ *Xylocoris flavipes* on a diet of *Liposcelis decolor* at different developmental stages and densities.

Predator	Prey Density (*N*)	Oviposition Efficiency (*N_o_*/*N*)		
			Prey Stage	
		Female	Male	Nymph
Adult♀ *X. flavipes*	2	1.47 ± 0.60 A	1.53 ± 0.63 A	1.53 ± 0.63 A
	6	0.59 ± 0.24 B	0.58 ± 0.24 B	0.59 ± 0.24 B
	15	0.24 ± 0.10 C	0.27 ± 0.11 BC	0.23 ± 0.10 C
	30	0.15 ± 0.06 C	0.16 ± 0.06 C	0.13 ± 0.05 C
	40	0.12 ± 0.05 C	0.12 ± 0.05 C	0.10 ± 0.04 C
	50	0.10 ± 0.04 C	0.10 ± 0.04 C	0.09 ± 0.04 C
	60	0.09 ± 0.03 C	0.08 ± 0.03 C	0.07 ± 0.03 C
	70	0.08 ± 0.03 C	0.07 ± 0.03 C	0.07 ± 0.03 C
	80	0.07 ± 0.03 C	0.07 ± 0.03 C	0.06 ± 0.02 C

Significant differences among oviposition efficiency values for different prey densities in the case of each prey stage diet are denoted by different letters (*p* ≤ 0.05, SAS, Tukey’s Honestly Significant Difference Test). There were no significant differences among oviposition efficiency values for diets of different prey stages in the case of each prey density.

**Table 4:** Per capita efficiency of conversion of ingested food resources (ECI) (mean ± SE) of adult♀ *Xylocoris flavipes* on a diet of *Liposcelis decolor* at different developmental stages and densities.

Predator	Prey Density (N)	Efficiency of Conversion of Ingested Food Resources (ECI)%		
		Prey Stage		
		Female	Male	Nymph
Adult♀ *X. flavipes*	2	149.07 ± 60.86 A	153.33 ± 62.60 A	156.11 ± 63.73 A
	6	59.79 ± 24.41 B	59.01 ± 24.09 B	60.93 ± 24.87 B
	15	27.43 ± 11.20 BC	28.76 ± 11.74 BC	26.49 ± 10.81 BC
	30	19.15 ± 7.82 C	18.72 ± 7.64 C	16.42 ± 6.71 C
	40	16.11 ± 6.58 C	14.51 ± 5.92 C	14.76 ± 6.03 C
	50	12.63 ± 5.16 C	13.60 ± 5.55 C	12.40 ± 5.06 C
	60	12.34 ± 5.04 C	11.12 ± 4.54 C	11.31 ± 4.62 C
	70	12.50 ± 5.10 C	11.37 ± 4.64 C	12.22 ± 4.99 C
	80	12.45 ± 5.08 C	11.61 ± 4.74 C	12.31 ± 5.02 C

Significant differences among ECI values at different prey densities in the case of each prey stage diet are denoted by different letters (*p* ≤ 0.05, SAS, Tukey’s Honestly Significant Difference Test). There were no significant differences among ECI values for diets of different prey stages in the case of each prey density.

## Data Availability

The original contributions presented in this study are included in the article. Further inquiries can be directed to the corresponding author.
